# Supplementary material for: The causal role of circulating inflammatory markers in osteoporosis: a bidirectional Mendelian randomized study
Source: Front Immunol. 2024 Jul 18;15:1412298. doi: 10.3389/fimmu.2024.1412298 (PMC11291241; doi:10.3389/fimmu.2024.1412298)
Supplement: Supplementary file 1 [file DataSheet_1.zip › supplementary material/CIM-OSTEOPOROSIS /GCST90274815.csv_leave_one_out.pdf]

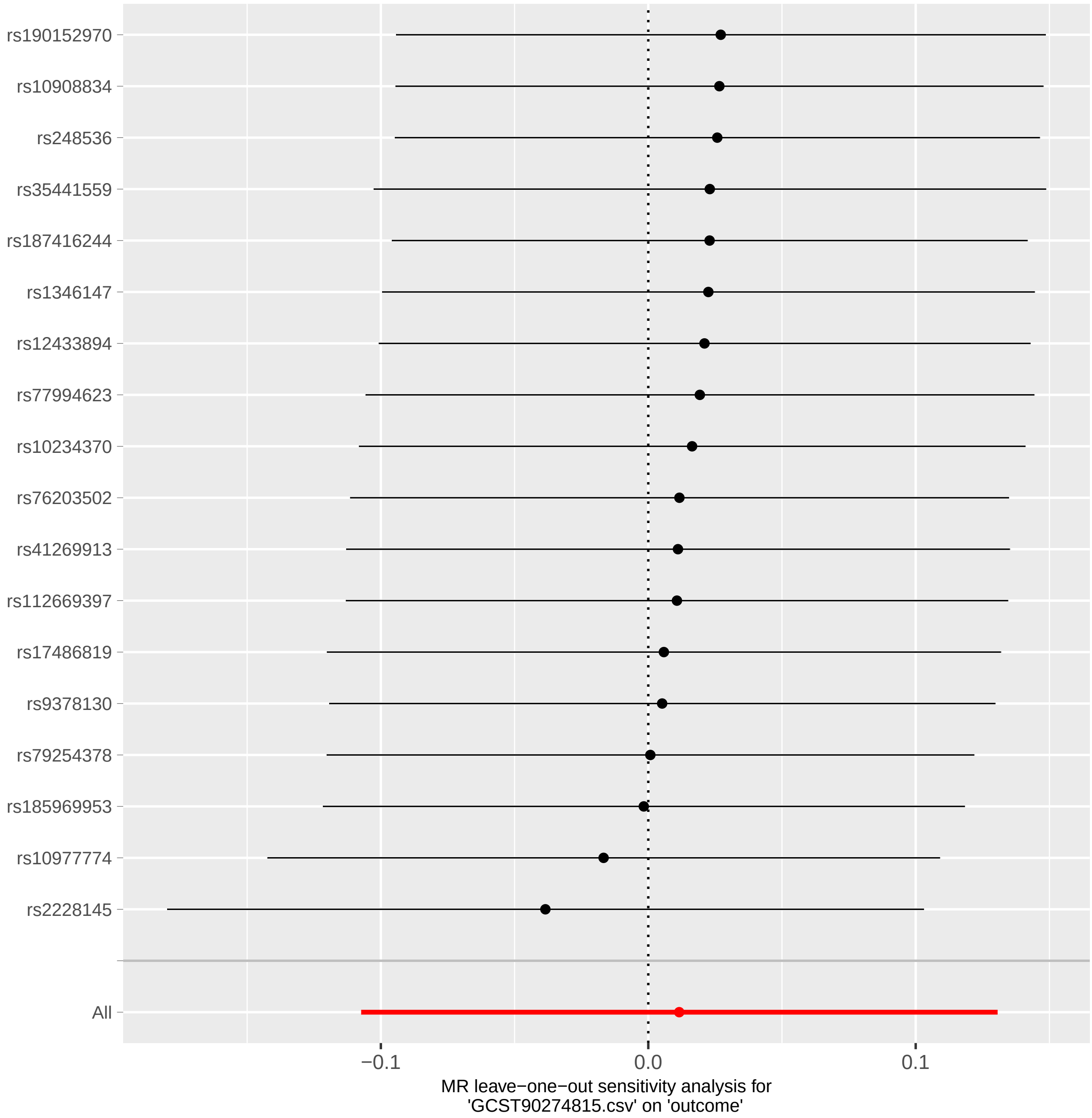
rs190152970
rs10908834
rs248536
rs35441559
rs187416244
rs1346147
rs12433894
rs77994623
rs10234370
rs76203502
rs41269913
rs112669397
rs17486819
rs9378130
rs79254378
rs185969953
rs10977774
rs2228145
All
−0.1
0.0
0.1
MR leave−one−out sensitivity analysis for
'GCST90274815.csv' on 'outcome'
